# Betulinic Acid Restricts Human Bladder Cancer Cell Proliferation In Vitro by Inducing Caspase-Dependent Cell Death and Cell Cycle Arrest, and Decreasing Metastatic Potential

**DOI:** 10.3390/molecules26051381

**Published:** 2021-03-04

**Authors:** So Young Kim, Hyun Hwangbo, Min Yeong Kim, Seon Yeong Ji, Da Hye Kim, Hyesook Lee, Gi-Young Kim, Sung-Kwon Moon, Sun-Hee Leem, Seok Joong Yun, Wun-Jae Kim, JaeHun Cheong, Cheol Park, Yung Hyun Choi

**Affiliations:** 1 Anti-Aging Research Center, Dong-eui University, Busan 47340, Korea; 14731@deu.ac.kr (S.Y.K.); hbhyun2003@naver.com (H.H.); ilytoo365@deu.ac.kr (M.Y.K.); 14602@deu.ac.kr (S.Y.J.); 14983@deu.ac.kr (D.H.K.); 14769@deu.ac.kr (H.L.); 2 Department of Molecular Biology, Pusan National University, Busan 46241, Korea; molecule85@pusan.ac.kr; 3 Department of Biochemistry, College of Korean Medicine, Dong-eui University, Busan 47227, Korea; 4 Department of Marine Life Sciences, School of Marine Biomedical Sciences, Jeju National University, Jeju 63243, Korea; immunkim@jejunu.ac.kr; 5 Department of Food and Nutrition, Chung-Ang University, Anseong 17546, Korea; sumoon66@cau.ac.kr; 6 Department of Biomedical Science, Dong-A University, Busan 49315, Korea; shleem@dau.ac.kr; 7 Department of Health Sciences, Dong-A University, Busan 49315, Korea; 8 Department of Urology, College of Medicine, Chungbuk National University, Cheongju 28644, Korea; sjyun@chungbuk.ac.kr (S.J.Y.); wjkim@chungbuk.ac.kr (W.-J.K.); 9 Division of Basic Sciences, College of Liberal Studies, Dong-Eui University, Busan 47340, Korea

**Keywords:** apoptosis, betulinic acid, bladder cancer, invasion, cell proliferation

## Abstract

Betulinic acid (BA) is a naturally occurring pentacyclic triterpenoid and generally found in the bark of birch trees (*Betula* sp.). Although several studies have been reported that BA has diverse biological activities, including anti-tumor effects, the underlying anti-cancer mechanism in bladder cancer cells is still lacking. Therefore, this study aims to investigate the anti-proliferative effect of BA in human bladder cancer cell lines T-24, UMUC-3, and 5637, and identify the underlying mechanism. Our results showed that BA induced cell death in bladder cancer cells and that are accompanied by apoptosis, necrosis, and cell cycle arrest. Furthermore, BA decreased the expression of cell cycle regulators, such as cyclin B1, cyclin A, cyclin-dependent kinase (Cdk) 2, cell division cycle (Cdc) 2, and Cdc25c. In addition, BA-induced apoptosis was associated with mitochondrial dysfunction that is caused by loss of mitochondrial membrane potential, which led to the activation of mitochondrial-mediated intrinsic pathway. BA up-regulated the expression of Bcl-2-accociated X protein (Bax) and cleaved poly-ADP ribose polymerase (PARP), and subsequently activated caspase-3, -8, and -9. However, pre-treatment of pan-caspase inhibitor markedly suppressed BA-induced apoptosis. Meanwhile, BA did not affect the levels of intracellular reactive oxygen species (ROS), indicating BA-mediated apoptosis was ROS-independent. Furthermore, we found that BA suppressed the wound healing and invasion ability, and decreased the expression of Snail and Slug in T24 and 5637 cells, and matrix metalloproteinase (MMP)-9 in UMUC-3 cells. Taken together, this is the first study showing that BA suppresses the proliferation of human bladder cancer cells, which is due to induction of apoptosis, necrosis, and cell cycle arrest, and decrease of migration and invasion. Furthermore, BA-induced apoptosis is regulated by caspase-dependent and ROS-independent pathways, and these results provide the underlying anti-proliferative molecular mechanism of BA in human bladder cancer cells.

## 1. Introduction

Bladder cancer, also known as bladder urinary tract carcinoma, is one of the most commonly diagnosed malignancies [1,2]. Bladder cancer occurs much more often in men than in women and is the second most common cancer in middle-aged and elderly men after prostate cancer. More than 70% of bladder cancer patients are non-muscle-invasive bladder cancer, and there is a high probability that it can be successfully treated through surgery. However, more than half of people with aggressive tumors that invade muscles develop metastasis and cause bladder cancer-related mortality [3,4,5]. Radical cystectomy is the standard therapy for bladder cancer, and various strategies such as radiation therapy, immunotherapy, chemotherapy, and their combination therapy are widely used in the clinic to treat bladder cancer [6,7]. Currently, drugs, such as doxorubicin, cisplatin, vincristine, and methotrexate are used for bladder cancer chemotherapy, but they have serious side effects and are ineffective or less tolerated [8,9]. Therefore, there is an urgent need for developing new therapeutic agents with low side effects and high efficiency for treating bladder cancer.

Natural products have played an important role in treating a variety of diseases [10,11,12]. Among them, many anti-cancer drugs currently used clinically are derived from plants or are derivatives of natural products [13,14,15]. Betulinic acid (BA), a kind of naturally occurring pentacyclic triterpenoid, is widely distributed throughout the plant kingdom, and is particularly abundant in the bark of the birch tree (*Betula* sp.) [16,17,18]. In previous studies, BA has been reported to possess various biological activities, including anti-bacterial, anti-viral, anti-inflammatory, antioxidant, anti-thrombotic, anti-fibrotic, hepatoprotective, anti-angiogenic, anti-tumor effects [17,18,19,20,21]. Among these properties, anti-tumor action has recently received great attention, showing that the anti-proliferative mechanism of cancer cells induced by BA is complex and depends on the cancer cell type [22,23,24,25], without causing toxicity toward non-cancer cells [26,27]. For example, BA inhibited cell proliferation and induced apoptosis in the (gap 0/gap 1) G0/G1 phase of cell cycle progression in human cervical cancer, oral squamous cell carcinoma, breast cancer, leukemia cells, etc., [28,29,30,31,32]. However, BA induced apoptosis in certain myeloma, gastric, and lung cancer cell lines, arresting the cell cycle at the synthesis (S) or gap 2/mitosis (G2/M) phase [33,34,35,36]. In addition, BA-induced apoptosis was accompanied by autophagy, which was involved in protecting or promoting cell death against cancer cell proliferation [37,38,39,40,41]. Moreover, BA has also been reported to block metastasis by inhibiting the mobility and invasion of cancer cells [42,43,44,45,46,47]. However, although BA has the potential to inhibit the proliferation of human bladder cancer cells [48], to our knowledge, the underlying anti-cancer mechanism of BA and its associated molecular targets are rarely identified in bladder cancer cells. Therefore, in this study, we investigated the underlying molecular mechanisms involved in the effect of BA on the growth inhibition of human bladder cancer cells.

## 2. Results

### 2.1. BA Inhibits Cell Proliferation in Human Bladder Cancer Cells

**Figure 1:**
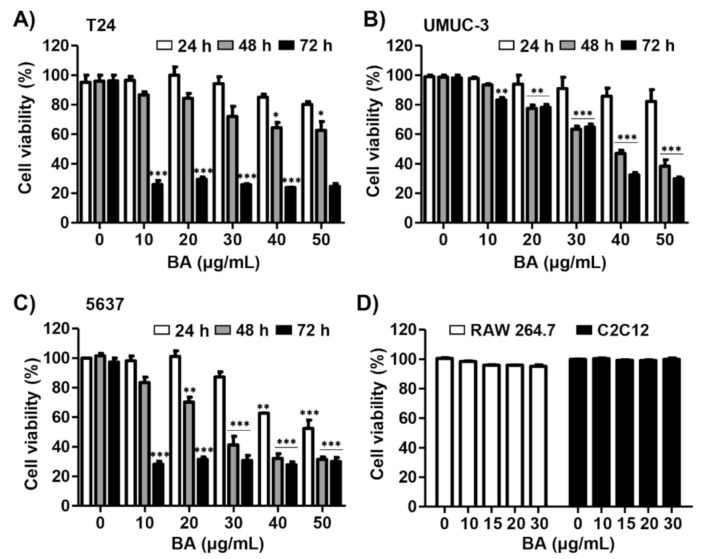
Betulinic acid (BA) inhibits cell viability in human bladder cancer cells. (**A**–**C**) Three bladder cancer cell lines T24, UMUC-3, and 5637 were treated with the indicated concentrations of BA for 24, 48, and 72 h. Cell viability was measured by the Cell Counting Kit-8 (CCK-8) assay. The data are expressed as mean ± standard deviation (SD) of three independent experiments. * *p* < 0.05, ** *p* < 0.01, and *** *p* < 0.001 vs. untreated control group. (**D**) RAW 264.7 macrophages and C2C12 myoblasts were treated with BA (0, 15, and 30 μg/mL) for 48 h. Cell viability was measured by the CCK-8 assay and is presented as the mean ± SD (*n* = 3).

Three human bladder cancer cell lines T24, UMUC-3, and 5637 were used to investigate the effect of BA on bladder cancer cell proliferation. As a result of Cell Counting Kit-8 (CCK-8) assay, the cell viability was inhibited in a dose and time-dependent manner in BA-treated cells (Figure 1A–C). Among the bladder cancer cell lines, 5637 cells were much more sensitive to BA than T24 or UMUC-3 cells under the same conditions. However, BA does not affect cell growth in normal cell lines including RAW 264.7 immortalized mouse macrophages and C2C12 immortalized mouse myoblasts (Figure 1D). These results suggest that BA has more potential effect on the suppression of cell proliferation of human bladder carcinoma cells than normal cells.

### 2.2. BA Induces Cell Death and Cell Cycle Dysregulation in Human Bladder Cancer Cells

**Figure 2:**
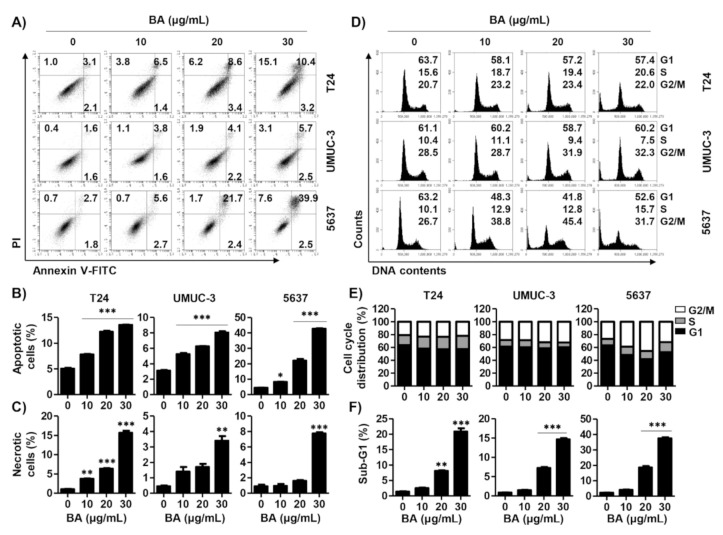
BA induces apoptosis, necrosis, and cell arrest in human bladder cancer cells. Cells were treated with BA (0, 10, 20, and 30 μg/mL) for 48 h. (**A**) Cells were collected and stained with annexin V-fluorescein isothiocyanate (FITC)/propidium iodide (PI), and then early apoptotic (annexin V^+^/PI^−^), late apoptotic (annexin V^+^/PI^+^), and necrotic (annexin V^−^/PI^+^) cells were measured by flow cytometry. (**B**,**C**) The proportion of apoptotic and necrotic cells were quantified. The percentage of apoptotic and necrotic cells are shown as the mean ± SD (*n* = 3). ** *p* < 0.01 and *** *p* < 0.001 vs. untreated control group. (**D**) The cell cycle distribution was detected using flow cytometer. (**E**,**F**) The percentages of cell cycle distribution and sub-G1 phase cells were quantified. The percentage of cell distribution and sub-G1 phase are shown as the mean ± SD (*n* = 3). ** *p* < 0.01 and *** *p* < 0.001 vs. untreated control group.

To examine whether BA-induced cytotoxicity is related to cell death, cells were stained with an annexin V-fluorescein isothiocyanate (FITC)/propidium iodide (PI) double staining. As shown in Figure 2A,B, annexin-V^+^ cells were increased in a dose-dependent manner, as compared to the control in three bladder cancer cell lines. Similar to the results of the CCK-8 assay, 5637 cells showed the highest rate of increase in the population of annexin-V^+^ cells among the three bladder cancer cell lines. In addition, the annexin-V^−^/PI^+^ cells, which is considered to be necrosis, were markedly elevated in bladder cancer cells upon exposure to 30 μg/mL BA for 48 h (Figure 2C). Next, to identify whether BA-induced cell death is related to cell cycle arrest, we performed the cell distribution analysis using PI staining. In 5637 cells, the cell population at G2/M phase was increased by BA, and the cell populations of G1 and S phase were decreased (Figure 2D,E). However, G/2M phase in 5637 cells slightly restored upon 30 μg/mL BA, which can result from DNA defects by severe cytotoxicity. In T24 and UMUC-3 cells, the cell distribution at G2/M phase tend to increase but there is no significance. Meanwhile, sub-G1 phase, an indicator of apoptotic cells, was markedly increased in a dose-dependent manner of BA (Figure 2F). These results suggest that BA induces apoptosis and involved partially G2/M phase arrest although it may differ slightly depending on the cell lines.

### 2.3. BA Regulates the Expression of G2/M Phaser-Related Proteins in Human Bladder Cancer Cells

**Figure 3:**
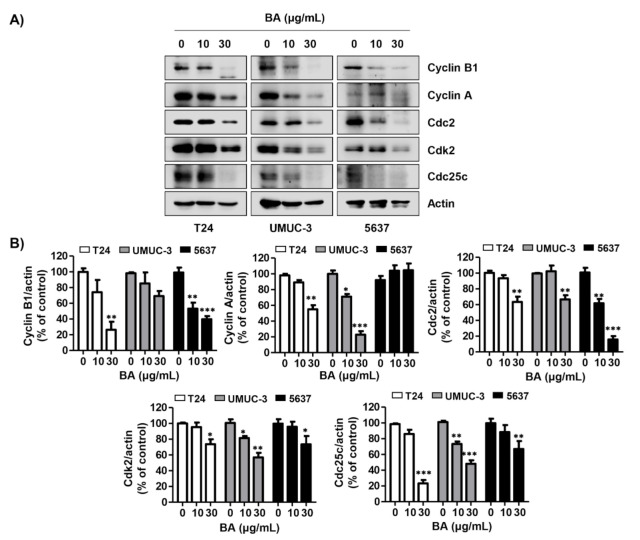
BA regulates the expression of G2/M phase-associated proteins in human bladder cancer cells. Cells were treated with BA (0, 10, and 30 μg/mL) for 48 h. (**A**) The G2/M phase-associated proteins (cyclin B1, cyclin A, Cdc2, Cdk2, and Cdc25c) were detected by Western blotting. Actin was used as a loading control. (**B**) Bar graphs indicate the relative band density in western blot analysis (*n* = 3). * *p* < 0.05, ** *p* < 0.001, and *** *p* < 0.0001 vs. untreated control group.

To determine whether BA influences the expression of G2/M phase-regulated proteins, we preformed Western blotting. Figure 3A,B shows that the expression of cyclin B1, cyclin A, cell division cycle 2 (Cdc 2), cyclin-dependent kinase 2 (Cdk 2), and Cdc25c was reduced by BA treatment in all three bladder cancer cells dose-dependently. These findings suggest that BA down-regulates the expression of G2/M-related proteins, and contributes to the apoptosis in human bladder cancer cells by BA-induced partial G2/M phase arrest.

### 2.4. BA Induces Loss of Mitochondrial Membrane Potential (MMP, ΔΨm) in Human Bladder Cancer Cells

**Figure 4:**
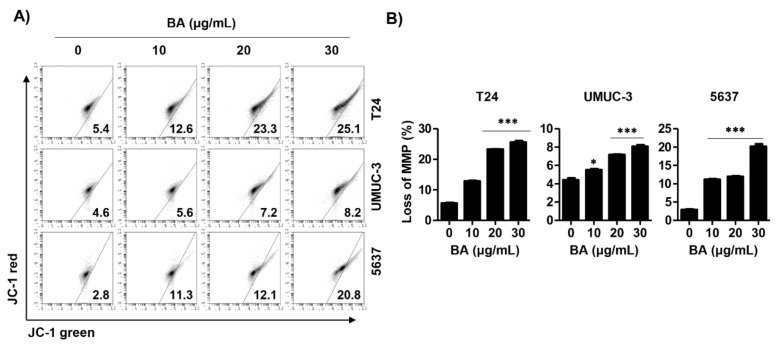
BA promotes mitochondrial dysfunction in human bladder cancer cells. Cells were treated with BA (0, 10, 20, and 30 μg/mL) for 48 h. (**A**) Cells were collected, stained with 5,5′6,6′-tetrachloro-1,1′,3,3′-tetraethyl-imidacarbocyanine iodide (JC-1) solution, and JC-1 green levels were analyzed using a flow cytometry. (**B**) The percentages of depletion of mitochondrial membrane potential (MMP) were quantified. Data are expressed as the mean ± SD (*n* = 3). * *p* < 0.05 and *** *p* < 0.001 vs. untreated control group.

**Figure 5:**
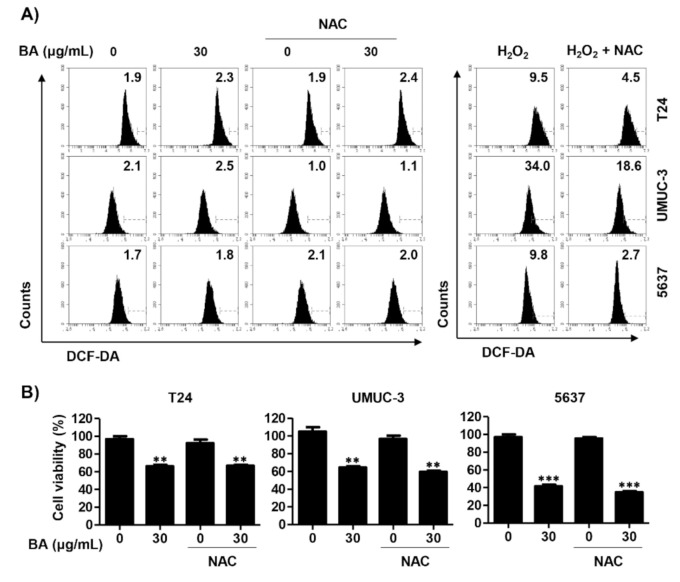
BA is not involved in reactive oxygen species (ROS) production in human bladder cancer cells. (**A**) Cells were pre-treated with or without *N*-acetylcysteine (NAC, 10 mM) for 1 h, and then cells were treated with BA (30 μg/mL) for 1 h or H_2_O_2_ (1 mM) for 15 min. Cells were stained with 5,6-carboxy-2′,7′-dichlorodihydrofluorescein diacetate (DCF-DA) for 20 min and were measured by flow cytometry. (**B**) Cells were pre-treated with or without NAC (10 mM) for 1 h, and then cells were treated with BA (0 and 30 μg/mL) for 48 h. Cell viability was measured using the CCK-8 assay. Each bar represents the mean ± SD of three independent experiments. ** *p* < 0.01 and *** *p* < 0.001 vs. untreated control group.

Based on the results that BA induced apoptosis in human bladder cancer cells, we assessed whether BA-induced cell death is involved in mitochondrial dysfunction. As shown in Figure 4A,B, 5,5′6,6′-tetrachloro-1,1′,3,3′-tetraethyl-imidacarbocyanine iodide (JC-1) green levels, an indicator of depletion of MMP (*ΔΨm*), were significantly increased following BA treatment in all three human bladder cancer cells. This result suggests that BA-induced apoptosis is associated with mitochondrial dysfunction.

### 2.5. BA-Mediated Apoptosis Is Not Associated with Reactive Oxygen Species (ROS) Production in Human Bladder Cancer Cells

Next, we investigated whether BA-induced mitochondrial dysfunction is associated with ROS production, because mitochondria are an important source of ROS that may trigger mitochondrial permeability transition pore induction [49]. ROS levels were investigated using 5,6-carboxy-2′,7′-dichlorodihydrofluorescein diacetate (DCF-DA) dye. As a result, we found that BA did not affect intracellular ROS levels in all three bladder cancer cells. To validate whether ROS works in these cells, we evaluated the effect of *N*-acetylcysteine (NAC), a ROS scavenger, on hydrogen peroxide-induced oxidative stress in the same cell lines. Our result showed that H_2_O_2_ treatment greatly increased intracellular ROS levels, whereas it was markedly suppressed by pre-treatment of NAC in all three cell lines (Figure 5A). In addition, BA-induced cell viability inhibition was not restored by NAC pre-treatment (Figure 5B). Therefore, these results verify that BA-induced cell death is not associated with ROS production in bladder cancer cells.

### 2.6. BA Induces Caspase-Dependent Apoptosis in Human Bladder Cancer Cells

**Figure 6:**
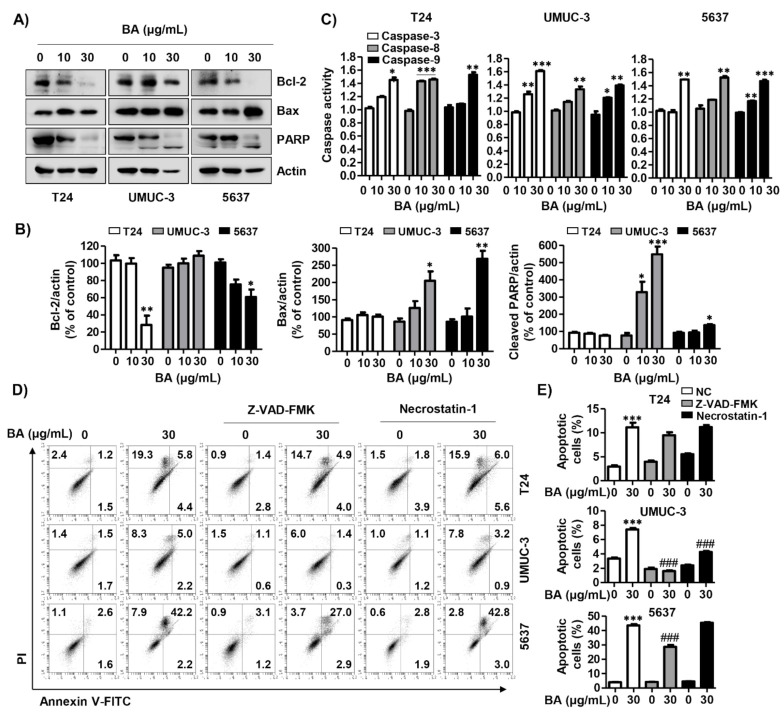
BA-induced apoptosis is associated with caspase activation in human bladder cancer cells. Cells were treated with BA (0, 10, and 30 μg/mL) for 48 h, and then cells were harvested. (**A**) The expression of mitochondrial-associated apoptotic proteins (Bcl-2 and Bax) and PARP were detected using Western blot analysis. Actin was used as loading control. (**B**) Bar graphs indicate the relative band density in western blot analysis (*n* = 3). * *p* < 0.05, ** *p* < 0.001, and *** *p* < 0.0001 vs. untreated control group. (**C**) The activities of caspase-3, -8, and -9 were measured by caspase colorimetric assay kits. Each bar represents the mean ± SD of three independent experiments. * *p* < 0.05, ** *p* < 0.001, and *** *p* < 0.0001 vs. untreated control group. (**D**) Cells were pre-treated with or without benzyloxycarbonyl-Val-Ala-Asp (OMe) fluoromethylketone (Z-VAD-FMK), which is pan-caspase inhibitor, or necrostatin-1, which is necrosis inhibitor, for 1 h, and then cells were treated with BA (0 and 30 μg/mL) for 48 h. Cells were collected, stained with annexin V-FITC/PI, and then apoptotic cells (annexin V^+^) and necrotic cells (PI^+^) were analyzed using flow cytometry. (**E**) The changed the proportion of apoptotic cells by treatments were quantified. Data are expressed as the mean ± SD (*n* = 3). *** *p* < 0.0001 vs. untreated control group and ### *p* < 0.0001 vs. BA-treated group.

Based on the results that BA led to MMP (*ΔΨm*) loss, we assessed the expression of mitochondrial-mediated apoptotic proteins following BA treatment. Figure 6A,B indicated that BA down-regulated the expression of anti-apoptotic Bcl-2 protein, while up-regulating the expression of pro-apoptotic proteins including Bcl-2-accociated X protein (Bax) and cleaved-poly-ADP ribose polymerase (PARP). However, BA did not change the expression of cleaved-PARP in T24 cells and BCl-2 in UMUC-3 cells. In addition, we identified whether BA-induced apoptosis was associated with the activation of caspase cascade, and the results showed that BA increased caspases-3, -8, and -9 activities in all three cell lines (Figure 6C). To confirm the role of caspase in BA-induced apoptosis, we evaluated the effect of benzyloxycarbonyl-Val-Ala-Asp (OMe) fluoromethylketone (Z-VAD-FMK), a pan-caspase inhibitor, through annexin V-FITC/PI staining analysis. As shown in Figure 6D,E, BA-induced apoptotic cells were significantly blocked by Z-VAD-FMK pre-treatment in UMUC-3 and 5637 cells. Although BA-induced apoptosis also decreased slightly in T-24 cells upon BA exposure, but there is no statistical significance. Furthermore, we verified the relation between necrosis and apoptosis on BA-induced cell death in human bladder cancer cells. The results showed that necrostatin-1, a necrosis inhibitor, significantly suppressed BA-induced apoptosis in UMUC-3 cells. These results suggest that BA-induced apoptosis was caspase-dependently regulated and associated with necrosis, which may differ depending on the cell lines.

### 2.7. BA Decreases Migration and Invasion of Human Bladder Cancer Cells

**Figure 7:**
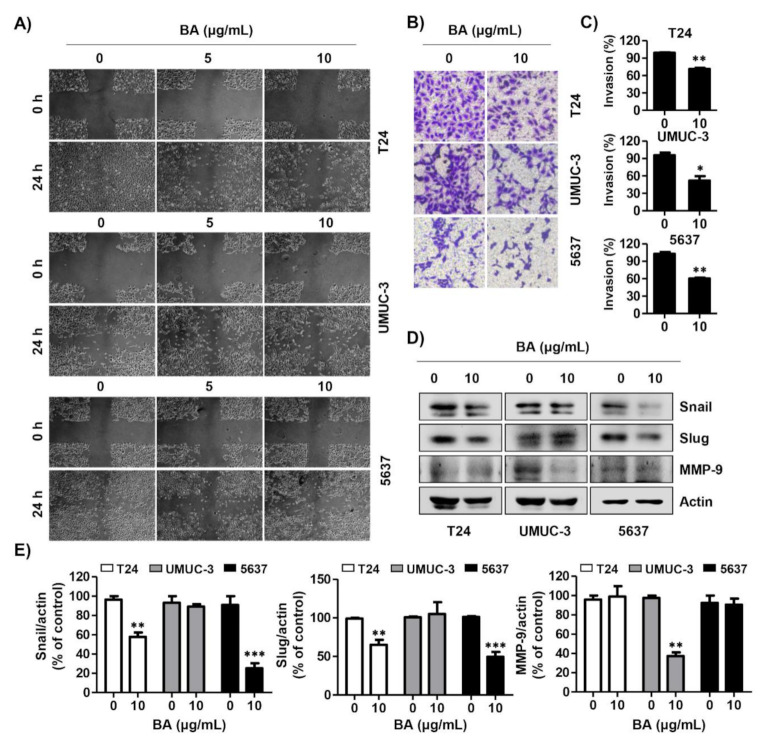
BA delays migration and invasion of human bladder cancer cells. (**A**) Cells were seeded, scratched, and then treated with BA (0, 5, and 10 μg/mL) for 24 h. The wound was measured using a phase-contrast microscope (magnification, 50×). (**B**) Cells were mixed BA with serum free medium and seeded in the upper Trans-well chamber, and then the medium containing 10% fetal bovine serum was added in the lower chamber. After 24 h of incubation, cells were washed, fixed, and stained with 0.1% crystal violet. The invasion cells were observed by a phase-contrast microscope (magnification, 50×). (**C**) The invading cells were calculated, as compared with the control cells. Data are expressed as the mean ± SD (*n* = 3). * *p* < 0.05 and ** *p* < 0.01 vs. control cells. (**D**) The expression of migration-associated proteins (Snail, Slug, and matrix metalloproteinase (MMP-9)) were detected using Western blot analysis. Actin was used as a loading control. (**E**) Bar graphs indicate the relative band density in Western blot analysis (*n* = 3). ** *p* < 0.001, and *** *p* < 0.0001 vs. untreated control group.

To investigate the effect of BA on migration and invasion in human bladder cancer cells, we performed wound healing and invasion assays. BA markedly decreased the wound-healing ability in T24, UMUC-3, and 5637 cells compared with the untreated cells (Figure 7A). In addition, BA significantly suppressed the cell invasion of all three cell lines (Figure 7B,C). Moreover, expression levels of the metastatic-related proteins (Snail, Slug, and matrix metalloproteinase (MMP)-9), were down-regulated by BA treatment. The expression of Snail and Slug were decreased in T24 and 5637 cells, but did not change in UMUC-3 cells, and the expression of MMP-9 was changed only in UMUC-3 cells (Figure 7D,E). These results suggest that BA delayed the migration and invasion in bladder cancer cells.

## 3. Discussion

Several studies have noted that BA has anti-tumor effects in various cancer cells, but, the underlying anti-cancer mechanism of BA in human bladder cancer cells is undisclosed until now [33,35,39,41,42,44,48]. Therefore, the identification of underlying anti-cancer molecular mechanism of BA is important to discovering new therapeutic agents for bladder cancer. Herein, we investigated the effect of BA on cell proliferation of three human bladder cancer cell lines including T24, UMUC-3, and 5637. Our finding suggested that BA has more potential effect on the suppression of cell proliferation of human bladder carcinoma cells than normal cells such as macrophages and myoblasts, and this result is similar to a previous study [26,27]. However, human bladder cancer cell lines T24, UMUC-3, and 5637 cells were observed to have somewhat different effects on cell death type upon BA treatment. T24 cells showed more higher rate of necrotic cells than apoptotic cells in response to BA exposure, whereas UMUC-3 cells slightly induced apoptosis. Meanwhile, BA-induced apoptosis markedly occurred in 5637 cells and BA-mediated necrosis was more less than apoptosis. These different results from three human bladder cancer cell lines may come from the genetic background gap between cell lines although derived from the same organ. In this respect, Ben-David et al. [50] established that 27 strains of the common breast cancer cell line MCF7 are highly genetically heterogeneous, which can result in drug response diversification. More recently, Liang et al. [51] demonstrated that mRNA and protein levels of cytochrome P450 family 27 subfamily a member 1 (CYP27A1) and androgen receptor were dissimilar between T24, UMUC-3, and 5637 cells. Furthermore, Shimizu et al. [52] reported that T24, UMUC-3, and 5637 cells differ in response to lipopolysaccharide and peptidoglycan. Additionally, Vasconcelos-Nóbrega et al. [53] suggested different responses of RAD001 treatment in bladder cancer cell lines including T24, 5637, and HT1376. In addition, although BA has anti-tumor effect in various cancer cells, accumulated evidence suggested that its cytotoxic effect may differ depending on the carcinoma origin and cell types [54,55]. The effective dose 50 (ED_50_) values of BA are 3 to 15 μg/mL and 5 to 16 μg/mL in medulloblastoma cell lines and glioblastoma cell lines, respectively [56]. Another study suggested that the half maximal inhibitory concentration (IC_50_) value of BA was 15 μg/mL in human lung cancer cells [57]. More recently, there is reported that the IC50 values of BA for pancreatic cancer PANC-1 cells and SW1990 cells were 21.5 μg/mL and 17.5 μg/mL, respectively [58]. Meanwhile, Hsu et al. [59] demonstrated that the IC_50_ values of BA was approximately 23 μg/mL in lung adenocarcinoma H1299 cells. In the present study, our finding showed that the IC_50_ values of BA were 33.2 μg/mL and 28.5 μg/mL in bladder cancer UMUC-3 and 5637 cells, respectively. Although the present study has limitation that we could not confirm the cytotoxic effect of BA in human bladder non-carcinoma cells, our present findings proved the first evidence that BA restricts human bladder cancer cells proliferation by inducing apoptosis and necrosis. Furthermore, further studies on drug response diversification following the genetic background difference in BA-treated bladder cancer cells are required. Moreover, we found that the suppression of cell proliferation following BA treatment was involved in cell death, including apoptosis and necrosis, which is accompanied by sub-G1 phase and partial G2/M phase arrest although it may differ slightly depending on the cell lines. One of the strategies of numerous anti-cancer drugs is targeting the cell cycle phases and checkpoints [60]. The progression of the cell cycle has Gap1 (G1), synthesis (S), G2, and M phases, which are regulated by Cdks and cyclins, and G1/S and G2/M phase are important checkpoints [61,62]. For proliferation, cells enter the G1/S phase for DNA synthesis and centrosome duplication, followed by entering into the G2/M phase for growth, preparation for mitosis, and division [60]. During the G2/M transition, Cdc2/cyclin A activity is required for initiating prophase, and Cdc2/cyclin B complexes actively participate in and complete M phases [63,64]. In the present study, we found that cyclin A, cyclin B1, Cdc 2, and Cdk 2 were reduced by BA treatment in all three bladder cancer cells. In this respect, our finding suggests that BA led to apoptosis and necrosis in human bladder cancer cells due to partial cell cycle arrest at the sub-G1 phase and G2/M phase via down-regulation of cyclin A, cyclin B1, Cdc2, and Cdk2.

As is well-known, apoptosis can be divided into two pathways; extrinsic and intrinsic. The extrinsic pathway is triggered by binding of ligands such as Fas ligand (FasL) and tumor necrosis factor-related apoptosis-inducing ligand (TRAIL), into the death receptors, followed by caspase-8 is activated [65]. The intrinsic pathway is associated with mitochondria dysfunction and Bcl-2 family proteins, and activates caspase-9 when this pathway is initiated by apoptosis stimuli. Activated caspase-8 and -9 induces caspase-3 activation, and subsequently led to the cleavage of various substrates such as PARP [66]. Our results showed that BA induced the depletion of MMP (*ΔΨm*), up-regulated the expression of pro-apoptotic proteins, and promoted the activities of caspases in three human bladder cancer cells. In addition, this finding showed that BA-induced apoptosis was caspase-dependently regulated and associated with necrosis, which may differ depending on the cell lines. Based on these findings, we considered that BA suppressed the proliferation of bladder cancer cells, which result from mitochondrial-mediated apoptosis, dependent of caspase. Interestingly, BA did not affect intracellular ROS levels in all three bladder cancer cells, although numerous studies have established that oxidative stress is a major cause of apoptosis by mitochondrial dysfunction [67,68]. Nevertheless, a few studies reported that cancer cell growth is inhibited by apoptosis via a ROS-independent pathway involving mitochondrial dysfunction [69]. Sharma et al. [69] reported anti-diabetic metformin as an anti-cancer agent inhibits proliferation of human breast cancer-derived cell lines by a ROS-independent apoptosis. Along with Sharma’s findings, our finding suggested that BA has anti-proliferative activity in bladder cancer cells through mitochondria-mediated intrinsic apoptotic pathway independently of ROS.

The metastasis and invasion are major abilities for cancer progression, thus that is a potential target for treating cancer [70]. Among the bladder cancer patients, approximately 25% of patients have muscle invasive disease caused by metastasis that essentially is responsible for aggressiveness, high grade disease, and mortality [6,71]. Accumulated evidences have established that epithelial-mesenchymal transition (EMT) progression is a key event in metastasis and invasion of tumor including bladder cancer [72,73,74]. EMT can be triggered by the various signaling pathways, and result in activation of the transcriptional factors including Snail, Slug, Zeb1, and Twist1, and subsequently lead to migration and invasion by up-regulation of MMP-2 and -9 [72,75,76]. One study reported that gigantol, a bibenzyl compound, inhibited the metastasis in bladder cancer cells through Wnt/β-catenin signaling [73], and another study showed that MiR-22 suppressed EMT progression through Snail and mitogen-activated protein kinase 1/Slug/vimentin [74]. In the present study, we found that BA suppressed migration and invasion in all three bladder cancer cells, as well as down-regulated the expression of the metastatic-related proteins including Snail, Slug, and MMP-9.

**Figure 8:**
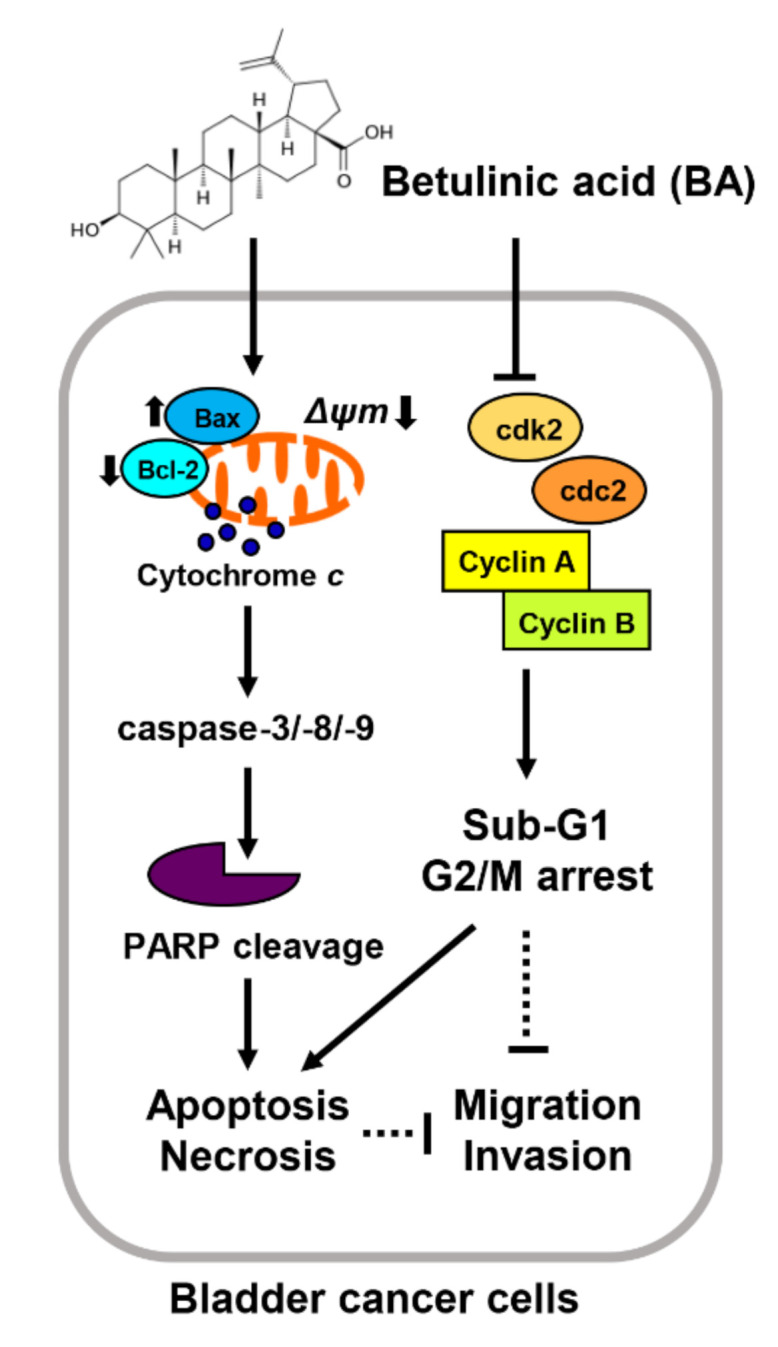
Proposed mechanism of cell proliferation effects of BA in human bladder cancer cells. BA induces apoptosis and necrosis, as well as delay of cell cycle at G2/M phase although it may differ slightly depending on the cell lines. Furthermore, BA results in mitochondrial dysfunction caused by MMP (*ΔΨm*) loss, which causes activation of mitochondrial-related intrinsic apoptosis regulators including Bax, caspases, and PARP cleavage. In addition, BA-induced apoptosis is regulated caspase dependently, but ROS independently in bladder cancer cells. Moreover, BA decreases migration and invasion abilities in bladder cancer cells.

Overall, this study has demonstrated that BA plays as a suppressor of bladder cancer cell growth by inducing apoptosis and necrosis, which is accompanied by sub-G1 phase and partial G2/M phase arrest although it may differ slightly depending on the cell lines. In addition, our findings suggested that BA have anti-metastatic and anti-proliferative activities via mitochondria-mediated intrinsic apoptotic pathway, dependent of caspase (Figure 8). Although further studies on drug response diversification following the genetic background difference in BA-treated bladder cancer cells are required, our results extend the understanding of anti-proliferative mechanism of BA and suggest that it may be potentially used for the prevention or treatment of human bladder cancer.

## 4. Materials and Methods

### 4.1. Cell Culture and BA Preparation

The human bladder cancer cell lines (T24, UMUC-3, and 5637) and normal cell lines (RAW 264.7 and C2C12 cells) were purchased from the American Type Culture Collection (Manassas, VA, USA). T24 and 5637 cells were cultured in Roswell Park Memorial Institute (RPMI) 1640 medium, and UMUC-3 and C2C12 cells were cultured in Dulbecco’s modified Eagle’s medium (DMEM) with 10% fetal bovine serum (FBS) and 1% penicillin/streptomycin (WELGENE Inc., Gyeongsan, Korea). RAW 264.7 cells were cultured in complete DMEM without penicillin/streptomycin. All cells were incubated at 37 °C in a humidified 5% CO_2_ atmosphere. BA was purchased from Sigma-Aldrich Chemical Co. (St. Louis, MO, USA), and was dissolved in dimethyl sulfoxide (Sigma-Aldrich Chemical Co.) to a final concentration of 10 mg/mL.

### 4.2. Cell Viability Assay

Cell viability was measured by the CCK-8 assay (Abcam, Cambridge, UK). Cells were seeded into the 6-well plates and treated with various concentrations of BA for 24 h and 48 h, and then added with 100 µL of the CCK-8 solution. After 30 min, the medium was transferred to the 96-well plates and measured at 450 nm using an enzyme-linked immunosorbent assay (ELISA) reader (VERSA Max, Molecular Device Co., Sunnyvale, CA, USA).

### 4.3. Cell Death Mode Detection

Cell death mode was detected by FITC-Annexin V and PI staining (BD Biosciences, San Diego, CA, USA). Cells were seeded into 6-well plates and treated with various concentrations of BA in the presence or absence of Z-VAD FMK or necrostatin-1. After 48 h, the cells were washed with PBS and collected by 0.05% trypsin- ethylenediaminetetraacetic acid (EDTA, WELGENE Inc.). Cells were centrifuged at 300 rpm for 5 min and then the supernatant was removed and double-stained with FITC-Annexin V and PI for 15 min, as previously described [77]. Cell death mode was analyzed by a flow cytometer (Accuri C6, BD Sciences) at the Core-Facility Center for Tissue Regeneration (Dong-eui University, Pusan, Korea). To exclude doublets cells, a forward scatter area (FSC-A) vs. side scatter area (SSC-A) density plot was used before analysis.

### 4.4. Cell Cycle Analysis

The cell cycle distribution was detected using a cycletest plus DNA reagent kit (BD Sciences) in accordance with the manufacturer’s instructions. Briefly, cells were seeded into the 6-well plates and treated with various concentrations of BA. After 48 h, cells were collected and re-suspended in solution for 30 min, as previously described [78].

### 4.5. Measurement of ROS Production

ROS production was detected by DCF-DA (Sigma-Aldrich Chemical Co.) staining as previously described [79]. After treatment with BA for 48 h in with or without NAC (Sigma-Aldrich Chemical Co.), cells were stained with 10 μM DCF-DA at 37 °C for 20 min. Continually, cells were harvested and measured using a flow cytometer (Accuri C6, BD Sciences).

### 4.6. Western Blot Analysis

Cells were treated with different concentrations of BA for 48 h, and then were harvested and total protein was extracted using lysis buffer as previously described [80]. Equal lysates were separated by sodium-dodecyl sulfate-polyacrylamide gel electrophoresis, and then transferred to polyvinylidene fluoride membranes (Millipore, Bedford, MA, USA). Subsequently, the membranes were blocked with 5% bovine serum albumin (Sigma-Aldrich Chemical Co.) for 1 h, and incubated with the following primary antibodies at 4 °C overnight: MMP-9 (Abcam), Bax (ProteinTech Group, Inc., Rosemont, IL, USA), PARP, cyclin B1, cyclin A, Cdc2, Cdk2, Cdc25c, Bcl-2, Snail, Slug, and Actin (Santa Cruz Biotechnology, Santa Cruz, CA, USA). In sequence, the membranes were washed and probed with horseradish peroxidase-conjugated secondary antibodies (Santa Cruz Biotechnology) at room temperature for 2 h. The signals were detected by enhanced chemiluminescent (Thermo Fisher Scientific, Waltham, MA, USA) and an image system (Vilber Lourmat, Torcy, France). The quantitative analysis of mean pixel density was performed by the ImageJ^®^ software.

### 4.7. MMP (ΔΨm) Analysis

The loss of MMP (*ΔΨm*) was measured by JC-1 staining (Sigma-Aldrich Chemical Co.). Cells were seeded with 6-well plates and treated with different concentrations of BA for 48 h. Cells were harvested and stained with JC-1 solution for 20 min, and then measured using a flow cytometer (Accuri C6, BD Sciences).

### 4.8. Caspase Activity

Caspase activities were measured using colorimetric assay kits (R&D Systems, Minneapolis, MN, USA) according to the manufacturer’s instructions. In brief, cells were treated with BA for 48 h, and then harvested and lysed in the lysis buffer for 10 min. Subsequently, cell lysates were centrifuged at 14,000 rpm for 30 min and equal amounts of proteins were incubated with the reaction buffer for 1–2 h at 37 °C. The caspase activities were measured using ELISA reader at 405 nm.

### 4.9. Wound Healing Assay

Cells were grown to complete confluence, and scraped with a 200 µL pipette tip as previously described [81]. Cells were incubated with or without BA in 0.5% serum-containing medium for 24 h. The width of wound area was captured by a phase-contrast microscope (Axio Scope, A1, Carl Zeiss, Oberkochen, Germany).

### 4.10. Transwell Invasion Assay

For invasion assay, Transwell chamber system (24 mm diameter, 8 μm pore size with polycarbonate membrane, Corning Costar Corp., Cambridge, MA, USA) was performed. Cells were suspended in serum-free medium, and then seeded into the upper chamber that was pre-coated with Matrigel (BD Sciences), and complete medium containing 10% FBS was added to the lower chamber. After 24 h, the lower surface of the upper chamber was fixed and stained with 0.5% crystal violet (Sigma-Aldrich Chemical Co.) for 30 min. The invasion cells were observed using a phase-contrast microscope (Carl Zeiss) as previously described [82]. The invasion distances were calculated using Image J analysis software (National Institutes of Health, Bethesda, MD, USA). The invading cells were calculated, and the relative invasive rate normalized to the control was indicated.

### 4.11. Statistical Analysis

GraphPad Prism 5.03 software (GraphPad Software, Inc., La Jolla, CA, USA) was used for data analysis. All experiments were presented as mean ± standard deviation (SD). Statistical evaluation for analysis was determined by a one-way analysis of variance (ANOVA), followed by Tukey’s post-hoc test. Two-tailed Student’s *t*-test was used for analyzing the rates of invasion compared with the control groups. *p* < 0.05 was considered statistically significant.

## 5. Conclusions

Our findings suggest that BA inhibits the proliferation of human bladder cancer cells, which is due to induction of apoptosis and necrosis, as well as delay of cell cycle at G2/M phase although it may differ slightly depending on the cell lines. Furthermore, BA results in mitochondrial dysfunction that is caused by MMP (*ΔΨm*) loss, and activated mitochondrial-related intrinsic apoptosis regulators including Bax, caspases and PARP cleavage. Importantly, BA-induced apoptosis is regulated caspase dependently, but ROS independently in bladder cancer cells. Moreover, BA decreases migration and invasion abilities in bladder cancer cells, and these results provide the underlying anti-proliferative molecular mechanism of BA for the treatment strategy in human bladder cancer.

## Data Availability

The data presented in this study are available within the article. Other data that support the findings of this study are available upon request from the corresponding authors.

## Sample Availability

Samples of the BA are available from the authors.
